# Tracking Systems for Virtual Rehabilitation: Objective Performance *vs.* Subjective Experience. A Practical Scenario

**DOI:** 10.3390/s150306586

**Published:** 2015-03-19

**Authors:** Roberto Lloréns, Enrique Noé, Valery Naranjo, Adrián Borrego, Jorge Latorre, Mariano Alcañiz

**Affiliations:** 1 Instituto Interuniversitario de Investigación en Bioingeniería y Tecnología Orientada al Ser Humano, Universitat Politècnica de València, Camino de Vera s/n, 46022 Valencia, Spain; E-Mails: vnaranjo@labhuman.com (V.N.); aborrego@labhuman.com (A.B.); jlatorre@labhuman.com (J.L.); malcaniz@labhuman.com (M.A.); 2 Servicio de Neurorrehabilitación y Daño Cerebral de los Hospitales NISA, Fundación Hospitales NISA, 46022 Valencia, Spain. E-Mail: enoe@comv.es; 3 Ciber, Fisiopatología Obesidad y Nutrición, CB06/03 Instituto de Salud Carlos III, Av. Sos Baynat s/n, Univesity of Jaume I, 12071 Castellón, Spain

**Keywords:** motion tracking, virtual reality, virtual rehabilitation, optical tracking, electromagnetic tracking, Kinect, stroke

## Abstract

Motion tracking systems are commonly used in virtual reality-based interventions to detect movements in the real world and transfer them to the virtual environment. There are different tracking solutions based on different physical principles, which mainly define their performance parameters. However, special requirements have to be considered for rehabilitation purposes. This paper studies and compares the accuracy and jitter of three tracking solutions (optical, electromagnetic, and skeleton tracking) in a practical scenario and analyzes the subjective perceptions of 19 healthy subjects, 22 stroke survivors, and 14 physical therapists. The optical tracking system provided the best accuracy (1.074 ± 0.417 cm) while the electromagnetic device provided the most inaccurate results (11.027 ± 2.364 cm). However, this tracking solution provided the best jitter values (0.324 ± 0.093 cm), in contrast to the skeleton tracking, which had the worst results (1.522 ± 0.858 cm). Healthy individuals and professionals preferred the skeleton tracking solution rather than the optical and electromagnetic solution (in that order). Individuals with stroke chose the optical solution over the other options. Our results show that subjective perceptions and preferences are far from being constant among different populations, thus suggesting that these considerations, together with the performance parameters, should be also taken into account when designing a rehabilitation system.

## 1. Introduction

Virtual reality (VR) applications provide a sensory perception of interactive synthetic environments that replace the real environment in a sensory channel [1]. VR systems can recreate safe and controlled virtual environments (VE), with the benefits of intensive, variable, and task-oriented experiences, while providing the proper sensory feedback to promote learning and motivation. For this reason, the application of VR techniques to the rehabilitation field, known as virtual rehabilitation (VRHB), has given rise to an increasing number of studies that report clinical benefits in the motor [2,3] and cognitive rehabilitation [4] of individuals with different pathologies.

To make the 3D interaction in the VE possible, it is necessary to establish the correspondence between the movements in the real and the virtual world. The interaction of the users can be supported by different devices, such as motion trackers, control devices (joysticks), eye trackers, data gloves, *etc.* Motion trackers or tracking systems locate the position of specific markers or sensors in the real world and transfer their 3D position to the VE [5,6]. Consequently, it is possible to know the location of extremities, joints, or other body parts by fixing these markers or sensors to those anatomical points. There are different tracking technologies depending on the physical principle in which they are based on: magnetic, optical, mechanical, inertial, hybrid, *etc.* Recent advances in computer vision have made human pose recognition from depth images reality [7]. Even though it cannot be considered as a tracking system in the literal sense, the skeleton tracking provides the 3D position of many body joints.

Regardless of the technology used, tracking systems can be classified according to their performance parameters: accuracy, jitter, latency, *etc.* [8]. The performance of the tracking systems directly affects the virtual experience, either due to physical or psychological factors [9]. For example, poor accuracy can cause incongruences between the movement and its representation, and/or the use of wearable sensors can also affect the natural movement patterns, *etc.* These incongruences with the natural interaction within the real world can affect the immersion in the VE. In addition, a bad performance can cause corrupted outcome measures and thus prevent their rigorous interpretation. This is especially critical in virtual motor rehabilitation applications because they are focused on the motor responses. Although some parameters have been provided by the corresponding manufacturers or by previous studies [10,11] (Table 1), some others still remain unexplored.

**Table 1:** Characteristics of the tracking systems under study. The table shows the characteristics of the tracking systems.

Characteristic	NaturalPoint^®^ OptiTrack^TM^ V100:R2	Polhemus™ G4™	Microsoft^®^ Kinect™
Measurements (cm)	Camera: 7.5 × 4.5 × 3.7 Marker: 4 (diameter)	Source: 10.2 × 10.2 × 10.2 Hub: 10.6 × 1.9 × 6.6 Sensor: 2.29 × 2.82 × 1.52	Camera: 7.5 × 4.5 × 3.7 (5.8 × 28.2 × 6.8 with the support base)
Weight (g)	Camera: 119.1 Marker: 8	Source: 725.7 Hub: 114.0 Sensor: 43.0	Camera: 590
Frequency (Hz)	100	120	30 (with 1 skeleton)
Latency (ms)	10	10 (in optimum conditions)	150–500 [12]
Resolution *	RGB: 640 × 480 (at 100 Hz) with 8 bits	-	RGB: 640 × 480 (at 30 Hz) with 8 bits Depth: 640 × 480 (at 30 Hz) with 11 bits
Field of view (°) *	Horizontal: 46 Vertical: 35 (Default lens, 4.5mm F#1.6)	-	Horizontal: 57 Vertical: 43
Wavelength (nm) *	850	-	850
Connections	Wireless	Sensor-Hub: Wired Hub-Source: Wireless (proprietary RF link at 2.4 GHz with frequency hopping architecture)	Wireless
Power supply	Camera: 5 V, 490 mA Marker: Passive	Source: 5 V, 1 A Hub: 5 V, 500 mA (rechargeable battery) Sensor: Passive	Camera: 12 V, 1.1 A
Cost ($)	1198 (including 2 cameras)	5250 (including 1 sensor)	249

* Resolution, field of view, and wavelength are parameters of the optical tracking systems.

When VR is used in rehabilitation, special requirements have to be considered. Besides the performance, VR technology has to satisfy not only the particular needs of the patients, but also the needs of the therapists, who traditionally have not a technological background [13]. When therapists are exposed to VR technologies, many questions arise: “Which tracking system should I use? What are their performance and limitations? How do they affect the virtual experience of my patients? How do they affect my work?”

The objectives of this study were twofold: (1) to study and compare the performance of three different tracking solutions (optical, electromagnetic, and skeleton tracking) in a practical scenario (a balance rehabilitation environment); and (2) to analyze the subjective responses of healthy and stroke individuals to the virtual experience when using the different technologies, and also their implications for therapists.

## 2. Experimental Section

### 2.1. Tracking Systems under Study

#### 2.1.1. Optical Tracking

**Figure 1:**
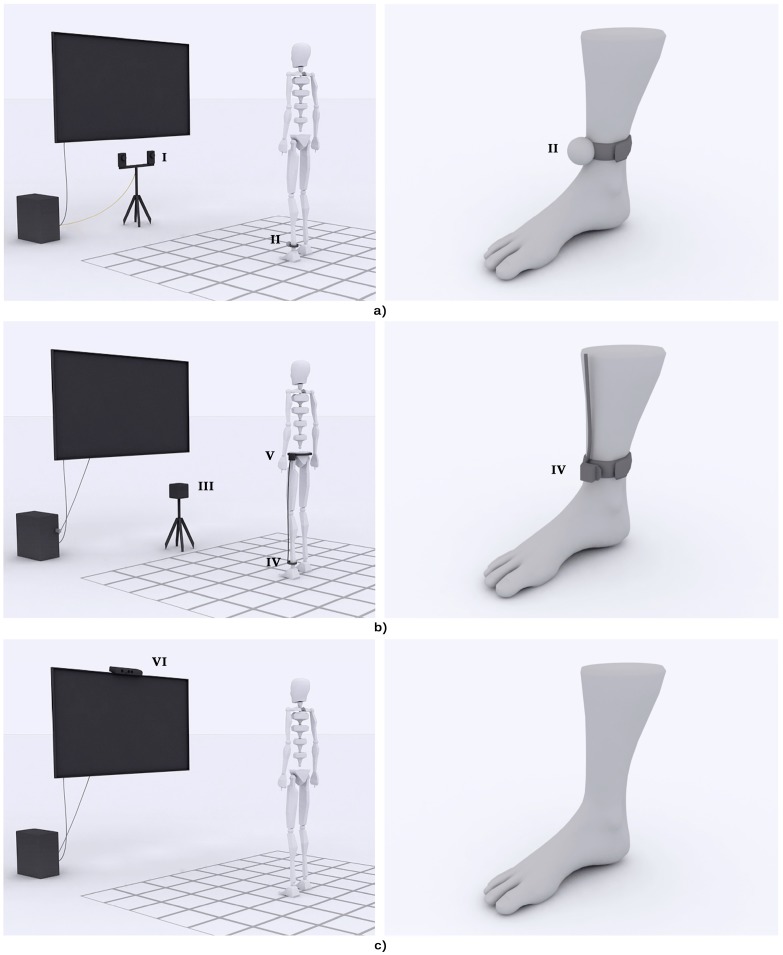
Setting of the tracking systems. Three different tracking systems were tested in the study. (**a**) The optical tracking solution used two cameras (I) and a passive reflective marker (II); (**b**) The electromagnetic tracking solution used a source (III) and a sensor (IV), wire connected to a hub (V); (**c**) The skeleton tracking solution used a depth sensor (VI).

To analyze the optical tracking performance, the OptiTrack™ FLEX:C120 (NaturalPoint^®^, Corvallis, OR, USA) cameras were chosen. In essence, a FLEX camera consists of an infrared (IR) sensor which is surrounded by a circular LED array. The LED array emits an IR light and the sensor captures the lighted scene. IR cameras capture the IR light reflected by the objects of a scene in a homologous manner to RGB cameras capture the (visible) light. Consequently, IR reflective spheres were used as markers. A spherical shape was chosen since it is invariant to spatial transforms. The 3D position of the markers was determined by photogrammetry, a technique that uses multiple view geometry to determine the 3D position of objects in a scene [14]. The projection of the marker in both cameras causes a disparity in their location that can be used to know the depth information. The 3D position of the markers can hence be estimated but not their orientation. In this study, we used the simplest configuration consisting of two cameras (Figure 1a).

#### 2.1.2. Electromagnetic Tracking

The electromagnetic tracking system used in this study was the G4™ (Polhemus^TM^, Colchester, VT, USA). The system consists of an emitter or source, few receivers or sensors, a hub, and a radiofrequency dongle (Figure 1b). In essence, the functioning of the G4™ is based on an electromagnetic field that is originated by the source and detected by the sensors. Depending on the amplitude and orientation of the detected electromagnetic field, it is possible to know the distance and orientation of the sensors [15]. The sensors are connected to a hub that transmits the 3D data to a dongle (plugged in a PC) and provides power to the sensors. This allows the wireless performance of the G4.

#### 2.1.3. Skeleton Tracking

Two of the most widely used off-the-shelf depth cameras, the Kinect^TM^ (Microsoft^®^, Redmond, WA, USA) and the Xtion cameras (ASUS^®^, Taipei, Taiwan), are based in the same chipset [16]. The pose estimation from single depth images is, however, different. In this study, the Kinect^TM^ was used (Figure 1c).

The depth sensor provides the Kinect^TM^ with its most interesting capabilities for motion tracking. It consists of an IR source and IR sensor distributed in the longitudinal axis of Kinect^TM^. The Kinect^TM^ tracking system is based on two main processes: the depth and pose estimation. The depth estimation processing uses light coding technology, a variation of the structured light technique that avoids synchronization. The IR sensor emits an IR beam that is scattered into a specific pattern of speckles that is continuously projected over the scene [17]. The depth sensor is calibrated by projecting the pattern on a flat surface at a known distance. Any object in the field of view of the depth sensor at a different distance produces a shift in the pattern. The depth data can therefore be estimated from the disparity between the original and the shifted pattern [18]. The pose estimation processing locally analyzes each pixel in the depth image and classifies it as part of the background or part of the user. In this last case, a decision forest trained with a large number of human poses is used to estimate the probability of each pixel of belonging to a body segment of a possible human pose. Finally, the algorithm estimates the position of 20 body joints that define the more plausible skeleton that fits the pose [7]. In this study, the Kinect for Windows SDK 1.7 was used.

### 2.2. Virtual Rehabilitation System

**Figure 2:**
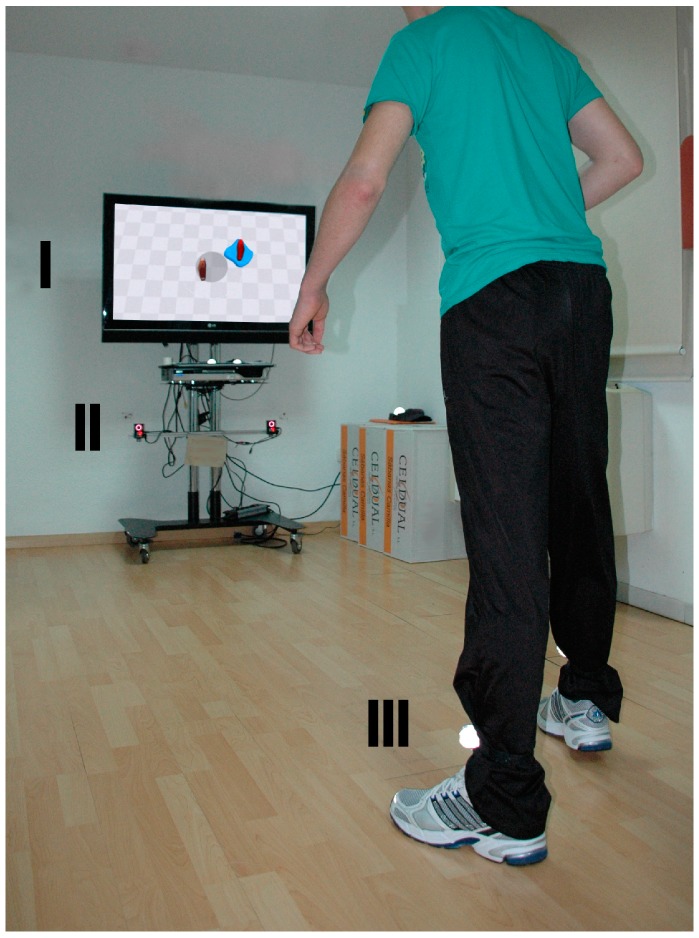
Participant interacting with the virtual rehabilitation system. The participant’s movements are tracked by two infrared cameras (II), which estimate the position of reflective markers attached to their ankles (III). The position of the markers are then transferred to the virtual environment, shown in a TV screen (I).

The VRHB system used in the experiment represents the participants’ feet in an empty scenario, which consists of a checkered floor that facilitates the depth perception with a central circle that represents the center of the VE. Different items rise from the floor around the circle. The objective of the task is to step on the rising items with the nearest foot while maintaining the other foot within the boundaries of the circle, and to recruit the extended foot afterwards [19,20] (Figure 2). The level of difficulty of the task can be defined by configuring the region of appearance, distance, size, lifetime and number of simultaneous items.

The hardware setting of the VRHB system consisted of a standard PC, a TV screen, and a tracking system. The PC, a Core^TM^ 2 Quad Q945 (Intel^®^, Santa Clara, CA, USA) @2.66 Hz with 4 GB of RAM and 1 GB video card running Windows® 7 (Microsoft^®^, Redmond, WA, USA), generated the VE. A 42'' LED screen provided audiovisual feedback. The movement of the participants’ feet was tracked and mimicked in the VE. All the aforementioned tracking technologies were allowed.

### 2.3. Study of the Performance

**Figure 3:**
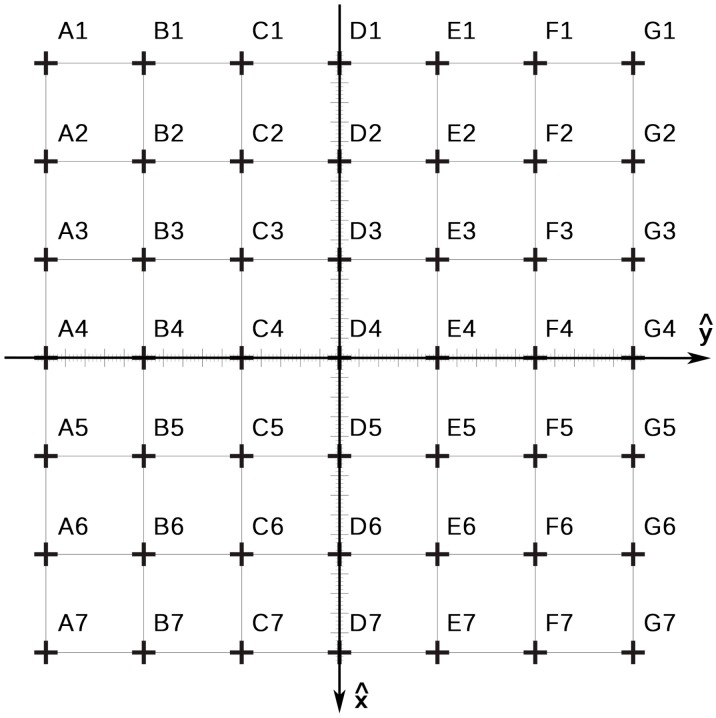
Measurement grid. A 6 × 6 grid with 25 cm × 25 cm squares covering an area of 1.5 m^2^ was used to measure the estimated position of the right ankle joint.

The present analysis focused on the accuracy and the jitter of the three tracking solutions described above. Since these parameters are far from being fixed, but mainly vary depending on the distance to the target (and on the environmental conditions), a practical scenario was designed to consider the effect of this parameter in the tracking performance. A 6 × 6 grid with 25 cm × 25 cm squares was designed and printed on a plastic sheet that was fixed to the floor (Figure 3). The grid covered an area of 1.5 m^2^.

The right ankle joint (tibiotalar) of an experimenter was fixed in all the intersection points of the grid (49 in total) using a cast. The position of the joint was estimated by the different tracking systems during 5 s. Accuracy (*e*) was estimated as the mean difference between the position of the target measured in the real world and its estimated position. The jitter (*j*) was defined as the maximum variation in the estimated position in the time interval as follows:

(1) $$e=\frac{1}{N}\overset{N}{\underset{i=1}{\sum }}|X_{i}-{\tilde{X}}_{i}|$$

(2) $$j=\left|\underset{N}{\text{max}}{\tilde{X}}_{i}-\underset{N}{\text{min}}{\tilde{X}}_{i}\right|$$

where *N* is the number of measurements provided by the tracking system in 5 s, which depends in turn on the update rate (or frequency) of each tracking system; $X_{i}$ is the real position of the targeted joint; and ${\tilde{X}}_{i}$ is its estimated position.

For the optical and electromagnetic systems, a sensor or a marker was fixed in the ankle joint of the user using a Velcro strip (Figure 1a,b). For the skeleton tracking, no sensor was used (Figure 1c). Close-fitting clothes were used to avoid possible displacements of the sensor or marker, and to avoid additional noise in the depth estimation of the Kinect^TM^. Clothing was pale and non-reflective to avoid false detections. The different tracking devices (sensors and sources) were placed at a distance that allowed their field of view or range to cover the area under study and were oriented along the x-axis (Figure 3). The optical setting was placed at a height of 50 cm and a distance of 225 cm from the grid. Both cameras were separated 40 cm. The electromagnetic source was placed at a height of 66.5 cm and a distance of 250 cm. Finally, the Kinect^TM^ was placed at a height of 80 cm and a distance of 250 cm.

The acquisition took place in a dedicated area of the physical therapy unit, previously cleared of reflective and electromagnetic materials that could interfere in both the optical and electromagnetic tracking, respectively. Preliminary tests showed abnormal performance of the electromagnetic tracking system due to distortion, presumably caused by the rebar. A software correction was done to compensate the error induced by this effect in any position of the space from previous estimations in known positions. A second grid, 8 × 8, which covered an area of 2 m^2^ was used to define these other positions. This calibration grid was placed over the estimation grid so that its center was displaced 12.5 cm in both x and y axes, thus avoiding overlapping of the intersection points of the calibration grid with those of the estimation grid. Acquisitions of the electromagnetic tracking were registered in all the intersection points of the calibration grid following the same protocol described above, and errors were estimated as the difference between the expected positions and the acquired values. The positions estimated by the electromagnetic tracking were corrected by bilinear interpolation [21], thus adding an offset calculated as a linear combination of the errors in the four surrounding points.

### 2.4. Study of Subjective Experiences

Three different configurations of the same VRHB system using the three different tracking systems were installed in the physical therapy area of a neurorehabilitation unit in a large metropolitan hospital. The experiences with the three tracking systems of healthy individuals, individuals with stroke, and therapists were collected through two ad-hoc questionnaires (A and B). Questionnaire A collected the experiences of both healthy individuals and individuals with stroke. Questionnaire B collected the experiences of therapists. The first four questions of the questionnaires A and B assessed the impressions of the three groups about the same features. The last question of both questionnaires evaluated the order of preference of the tracking systems.

Questionnaire A consisted of eight items that assessed (1) fixation speed of the sensors/markers; (2) ease of the calibration; (3) accuracy of the represented movements; (4) robustness of the tracking; (5) comfort while wearing the sensors/markers; and (6) order of preference of the tracking systems. Participants rated the first seven items on a 5-point Likert scale, where 1 means “very little/not at all” and 5 means “very much”. Responses to the last item were estimated as a percentage of preference.

Questionnaire B consisted of twelve items that assessed (1) fixation speed of the sensors/markers; (2) ease of the calibration; (3) accuracy of the represented movements; (4) robustness of the tracking; (5) ease of fixation of the markers/sensors, (6) insensibility of the performance to changes in the clinical setting; (7) ease of assistance; (8) maintenance of the tracking system; (9) working range of the tracking solution; (10) integration in the clinical setting; (11) value for money; and (12) order of preference. Again, participants rated the first eleventh items on a 5-point Likert scale. Responses to the last item were estimated as a percentage of preference.

#### 2.4.1. Healthy Individuals and Individuals with Stroke

The inclusion criteria in the healthy group were (1) age ≥55 and <80; and (2) absence of previously reported motor or cognitive limitations. Individuals with previous experience with VRHB systems were excluded. The inclusion criteria in the stroke group were (1) age ≥55 years old and <80 years old; (2) chronicity >6 months; (3) absence of severe cognitive impairment (Mini-mental state examination [22] cut-off >23); (4) able to follow instructions; (5) ability to maintain stride-standing position for 30 s without holding onto or assistance from another person as specified in the Brunel balance assessment (BBA), Section 3, level 7 [23]; and (6) Berg balance scale [24] score ≥ 41. The exclusion criteria were (1) individuals with previous experience with VRHB systems; (2) individuals with severe dementia or aphasia; (3) individuals whose visual or hearing impairment did not allow the possibility of interaction with the system; (4) individuals with hemispatial neglect; and (5) individuals with ataxia or any other cerebellar symptom. After inclusion/exclusion criteria the healthy group consisted of 19 individuals (12 males and 7 females, 60.8 ± 4.1 years old) and the stroke group consisted of 22 individuals (15 males and 7 females, 60.1 ± 7.0 years old). The stroke group included ischemic (*n* = 11) and haemorrhagic stroke (*n* = 11), and presented a chronicity of 272.4 ± 56.7 days.

All the participants interacted with the VRHB system a total of 45 min divided in 15-min sessions with each technology. The order in which the tracking systems were used was balanced in all the participants. The level of difficulty of the stepping task was configured to be within the motor skill of each participant, in such a way that the task could be performed. A physical therapist supervised all the sessions. After each session the participants filled the questionnaire A, rating their experience with the system. After the last session, the participants evaluated their order of preference (last item of the questionnaire).

#### 2.4.2. Professionals

The inclusion criteria in the therapists group were (1) physical therapy degree; and (2) ≥2 years of experience in neurorehabilitation. Therapists with previous experience with VRHB systems were excluded. A total sum of 47 physical therapists were working in one of the five hospitals that were part of the neurorehabilitation network. From them, 23 satisfied the criteria to participate in the study. The final sample consisted of 14 therapists (6 males and 8 females, 31.8 ± 2.4 years old) who accepted to be included in the study. All the participants belonging to the therapists group supervised and guided the virtual training of individuals with stroke who were attending a neurorehabilitation program in the unit during 45 sessions. The therapists used each system during 15 sessions in randomized order. The randomization schedule was computer-generated using a basic random number generator. Therapists were uninformed of the costs of the tracking systems during the entire study. After the 45 sessions, the therapists filled in the questionnaire B for the optical, electromagnetic, and skeleton tracking. Therapist were informed about the costs of each system just before rating the value for money of each system.

The VRHB therapy had already been integrated in the physical therapy program of the neurorehabilitation unit before this study and patients were included in the program according to their particular condition and expected clinical benefits. The existing VRHB system used the optical tracking system. Two more settings using the electromagnetic and the skeleton tracking system were installed in the unit.

### 2.5. Statistical Analysis

Demographical comparisons among groups were performed with independent sample t-tests and Chi-squared or Fisher exact tests, as appropriate. Repeated measures analyses were performed using the non-parametric Friedman test (χ^2^, p values) to determine within-group differences between tracking systems (OptiTrack^TM^ FLEX:C120 (NaturalPoint^®^), G4^TM^ (Polhemus^TM^), and Kinect^TM^ (Microsoft^®^)). When the Friedman test yielded a significant effect (*p* < 0.05), post hoc analysis was performed using a Wilcoxon signed-rank test for pairwise comparisons between tracking systems. The α level was set at 0.05 for all analyses. All analyses were computed with SPSS for Mac, version 20 (SPSS Inc., Chicago, IL, USA).

## 3. Results and Discussion

### 3.1. Objective Performance

Results of the acquisition are shown in Table 2.

**Table 2:** Performance results. The table shows the accuracy and jitter values estimated in the intersection points of the grid. Results are defined in terms of mean and standard deviation.

Characteristic	NaturalPoint^®^ OptiTrack^TM^ V100:R2	Polhemus^TM^ G4^TM^	Microsoft^®^ Kinect^TM^
Working Range (m^2^)	2.6	2.2	3.1
**Accuracy (cm)**			
X coordinate	0.6 ± 0.4	5.9 ± 3.0	0.9 ± 0.6
Y coordinate	0.6 ± 0.4	2.3 ± 2.4	2.4 ± 1.4
Z coordinate	0.4 ± 0.2	8.3 ± 1.8	1.0 ± 1.0
Total	1.1 ± 0.4	11.0 ± 2.4	2.9 ± 1.4
**Jitter (cm)**			
X coordinate	0.4 ± 0.3	0.2 ± 0.1	1.3 ± 0.7
Y coordinate	0.1 ± 0.1	0.1 ± 0.1	0.3 ± 0.3
Z coordinate	0.1 ± 0.0	0.2 ± 0.1	0.6 ± 0.5
Total	0.4 ± 0.3	0.3 ± 0.1	1.5 ± 0.9

The optical tracking system provided the most accurate results (1.1 ± 0.4 cm) even though only two markers were used. More complex solutions using more than two cameras and two markers could have been used to increase the accuracy [25]. Different motion tracking systems use this technology to precisely track human movements [26]. However, this could have also led to a more difficult integration in the clinical setting. In addition, even though the distortion (radial and tangential) of both cameras were corrected [14], better optics and higher resolution could have led to better performance. The mean jitter value of the optical solution was 0.4 ± 0.3 cm, which can be considered as reasonable in absolute terms, in spite of the fact that it is nearly 50% of its accuracy. Since the tracking area was defined by the common field of view of both cameras, the setting of the optical solution required and area of 1.5 × 3.75 m (working range in x-axis from 2.25 to 3.75 m), which could exceed the space availability of standard clinical facilities. This distance can be decreased by slightly orienting the cameras one to each other, at the expense of increasing the complexity of the mathematical formulation to estimate the 3D position of the markers.

The skeleton tracking system provided an accuracy of 2.9 ± 1.4 cm, which is three times the error provided by the optical solution. The errors of this tracking system are mainly in the YZ plane, it is, in planes perpendicular to the Kinect™ axis. This is mainly due to the noise derived from the depth estimation technique [17]. The use of this technique also affected the jitter, providing the highest value (1.5 ± 0.9 cm), and the working range, providing the most restrictive values (from 2.5 to 4 m in x-axis), even though this tracking system covered the largest area. All these facts can prevent its use for applications that require high accuracy, and/or in clinical facilities with limited space. Even though the position of the ankle joint was estimated in static condition, the accuracy of this solution while measuring joints in movement has been reported to have similar values [27]. A worse performance of the statistical estimation of the body segments, and of the following fitting of a biomechanical skeleton in the detected human shape could explain wrong pose estimations during movement.

The electromagnetic tracking solution provided the least accurate results (11.0 ± 2.4 cm), in spite of the correction of the electromagnetic distortion. The experiment showed that even though ferromagnetic devices as wheelchairs, physiotherapy plinths, canes, *etc.* caused small or no impact on the measures when they got closer to the tracker, the rebar on the floor severely affected its performance. The electromagnetic field generated by the source induced eddy currents in the rebar, which in turn generated electromagnetic fields that distorted the measures [28]. This effect was reduced by the software correction but to a limited extent. Different calibration grids, with different grid sizes, were tested with very similar results. In addition to the distortion, the area required for the stepping exercise, replicated in the study of the objective performance, exceeded the tracker reliable range. Therefore, the use of this tracking system in rehabilitation should be evaluated considering these two main limitations. However, the electromagnetic solution provided the best jitter values (0.3 ± 0.1 cm), which means that the location of the ankle joint, even less accurate, was the most stable.

### 3.2. Subjective Performance

**Figure 4:**
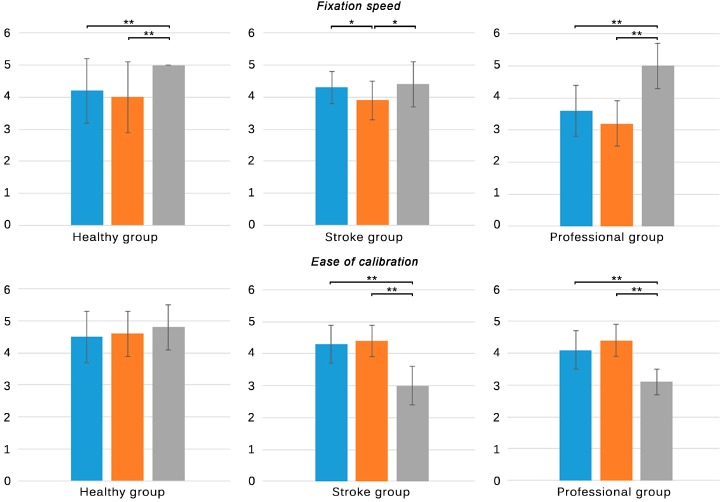

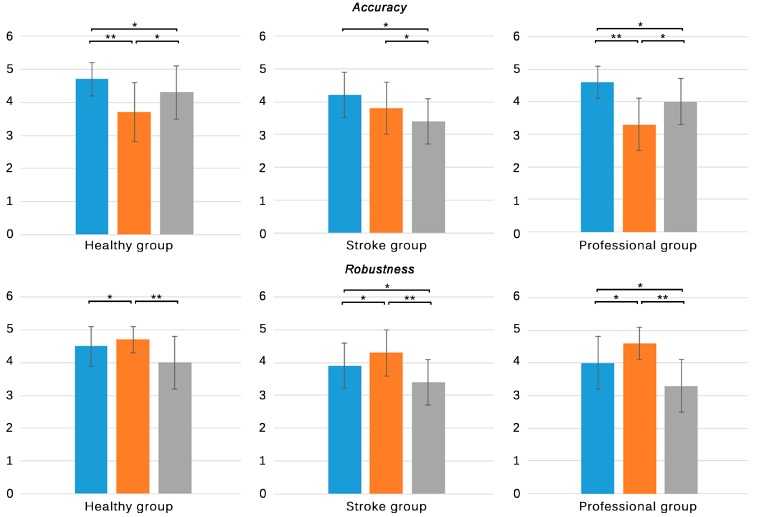
Subjective responses of all groups to the first four items of questionnaires **A** and **B**. **Blue**: NaturalPoint^®^ OptiTrack^TM^; **Orange**: Polhemus^TM^ G4^TM^; **Grey**: Microsoft^®^ Kinect^TM^. Only significant differences are stated. * *p* < 0.05, ** *p* < 0.001.

**Figure 5:**
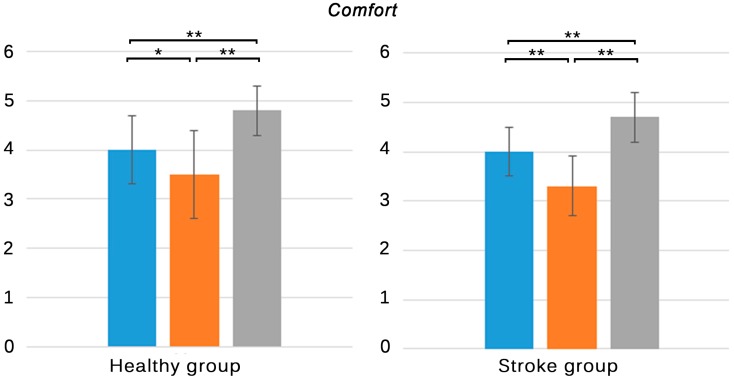
Subjective responses of healthy subjects and individuals with stroke to item five of questionnaire A. **Blue**: NaturalPoint^®^ OptiTrack^TM^; **Orange**: Polhemus^TM^ G4^TM^; **Grey**: Microsoft^®^ Kinect^TM^. Only significant differences are stated. * *p* < 0.05, ** *p* < 0.001.

**Figure 6:**
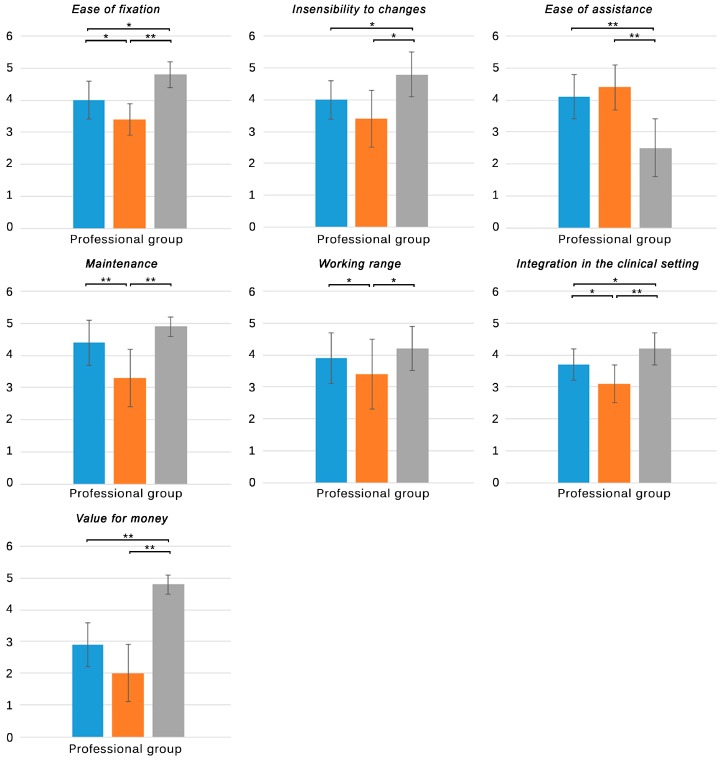
Subjective responses of therapists to items five to nine of questionnaire B. **Blue**: NaturalPoint^®^ OptiTrack^TM^; **Orange**: Polhemus^TM^ G4^TM^; **Grey**: Microsoft^®^ Kinect^TM^. Only significant differences are stated. * *p* < 0.05, ** *p* < 0.001.

**Figure 7:**
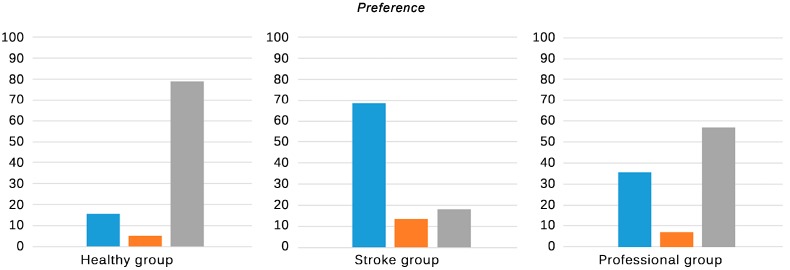
Subjective responses of all groups regarding their order of preference. **Blue**: NaturalPoint^®^ OptiTrack^TM^; **Orange**: Polhemus^TM^ G4^TM^; **Grey**: Microsoft^®^ Kinect^TM^. Only significant differences are stated. * *p* < 0.05, ** *p* < 0.001.

Results of the subjective experiences, as registered in the ad-hoc questionnaires, are shown in Table 3. Statistical significance relations are also depicted in Figure 4, Figure 5, Figure 6 and Figure 7.

**Table 3:** Subjective experiences. The table shows the scores of each group to the subjective questionnaires. Results are defined in terms of mean and standard deviation. Only significant differences are stated.

Issue	NaturalPoint^®^ OptiTrack^TM^	Polhemus™ G4^TM^	Microsoft^®^ Kinect^TM^	Significance
**Healthy, Stroke Individuals, and Professionals**				
A1/B1. Fixation speed of sensors/markers				
Healthy group	4.2 ± 1.0	4.0 ± 1.1	5.0 ± 0.0	O = G , K ** > O, K ** > G
Stroke group	4.3 ± 0.5	3.9 ± 0.6	4.4 ± 0.5	O * > G, O = K , K * > G
Professional group	3.6 ± 0.8	3.2 ± 0.7	5.0 ± 0.0	O = G, K ** > O, K ** > G
A2/B2. Ease of calibration				
Healthy group	4.5 ± 0.8	4.6 ± 0.7	4.8 ± 0.7	NS
Stroke group	4.3 ± 0.6	4.4 ± 0.5	3.0 ± 0.6	O = G , O ** > K, G ** > K
Professional group	4.1 ± 0.6	4.4 ± 0.5	3.1 ± 0.4	O = G , O ** > K, G ** > K
A3/B3. Accuracy				
Healthy group	4.7 ± 0.5	3.7 ± 0.9	4.3 ± 0.8	O ** > G, O * > K *, K * > G
Stroke group	4.2 ± 0.7	3.9 ± 0.8	3.4 ± 0.7	O = G, O * > K, G * > K
Professional group	4.6 ± 0.5	3.3 ± 0.8	4.0 ± 0.7	O ** > G, O * > K, K * > G
A4/B4. Robustness				
Healthy group	4.5 ± 0.6	4.7 ± 0.4	4.0 ± 0.8	G * > O, O = K, G ** > K
Stroke group	3.9 ± 0.7	4.3 ± 0.7	3.4 ± 0.7	G * > O, O * > K, G ** > K
Professional group	4.0 ± 0.8	4.6 ± 0.5	3.3 ± 0.8	G * > O, O * > K, G ** > K
**Healthy and Stroke Individuals**				
A5. Comfort				
Healthy group	4.0 ± 0.7	3.5 ± 0.9	4.8 ± 0.5	O * > G, K ** > O, K ** > G
Stroke group	4.0 ± 0.5	3.3 ± 0.6	4.7 ± 0.5	O ** > G, K ** > O, K ** > G
Professional group	-	-	-	-
**Professionals**				
B5. Ease of fixation				
Healthy group	-	-	-	-
Stroke group	-	-	-	-
Professional group	4.0 ± 0.6	3.4 ± 0.5	4.8 ± 0.4	O * > G, K * > O, K ** > G
B6. Insensibility to changes in the clinical setting				
Healthy group	-	-	-	-
Stroke group	-	-	-	-
Professional group	3.1 ± 0.6	3 ± 0.8	3.7 ± 0.5	O = G, K * > O, K * > G
B7. Ease of assistance				
Healthy group	-	-	-	-
Stroke group	-	-	-	-
Professional group	4.1 ± 0.7	4.4 ± 0.7	2.5 ± 0.9	O ** > K, G ** > K, O = G
B8. Maintenance				
Healthy group	-	-	-	-
Stroke group	-	-	-	-
Professional group	4.4 ± 0.7	3.3 ± 0.9	4.9 ± 0.3	O ** > G, O = K, K ** > G
B9. Working range				
Healthy group	-	-	-	-
Stroke group	-	-	-	-
Professional group	3.9 ± 0.8	3.2 ± 1.1	4.2 ± 0.7	O * > G, O = K, K * > G
B10. Integration in the clinical setting				
Healthy group	-	-	-	-
Healthy group	-	-	-	-
Professional group	3.7 ± 0.5	3.1 ± 0.6	4.2 ± 0.5	O * > G, K * > O, K ** > G
B11. Value for money				
Healthy group	-	-	-	-
Stroke group	-	-	-	-
Professional group	2.5 ± 0.5	2.3 ± 0.7	4.8 ± 0.3	K ** > O, K ** > G, G = O
**Healthy, Stroke Individuals, and Professionals**				
A8/B12. Preference (n, %)				
Healthy group	3 (15.8%)	1 (5.2%)	15 (79.0%)	-
Stroke group	11 (50%)	3 (13.6%)	8 (36.4%)	-
Professional group	4 (28.6%)	3 (21.4%)	7 (50%)	-

K = Microsoft^®^ Kinect^TM^, O = NaturalPoint^®^ OptiTrack^TM^, G4 = Polhemus^TM^ G4^TM^. Friedman with Wilcoxon as post-hoc. * *p* < 0.05, ** *p* < 0.001. Significance: >higher than, =same as.

All the groups evaluated their experiences with the three tracking systems regarding the fixation of the markers, ease of calibration, accuracy, and robustness (Figure 4). With regards to the time to fix the markers, all the groups reported the Kinect^TM^ as the least time consuming system, followed by the optical and the electromagnetic solution. Differences between the skeleton tracking and the other solutions were significant in the healthy and professional group (*p* < 0.001), who reported no significant differences between the optical and electromagnetic solution. The stroke group reported differences not only between the skeleton and electromagnetic solutions (*p* < 0.05) but also between the optical and electromagnetic tracking systems (*p* < 0.05). Despite the significant difference between the skeleton and the optical tracking reported by the healthy and professional group (0.8 and 1.4 in mean, respectively), individuals with stroke did not find this difference as relevant (0.1 in mean) (Table 3. A1/B1 item). The fixation speed of the electromagnetic sensors was reported to be the lowest by the three groups. Interestingly, the professionals evaluated it with the lowest score, which can be explained by the fact that they also had to be careful with the position of the wires to avoid tangles.

Regarding the ease of calibration, no significant differences between tracking systems were reported by the healthy group (Table 3. A2/B2 item). However, the stroke and professional group found the calibration for the skeleton tracking to be significantly more difficult than for the other systems (*p* < 0.001). While the calibration for the optical and electromagnetic system just consisted on being self-supported in standing position, a requirement which was considered in the inclusion/exclusion criteria that was satisfied by all the participants, the calibration of the Kinect^TM^ required the participants to move to be tracked. This fact made the task more difficult for individuals with stroke who presented motor impairments, as reported by clinicians and themselves. It has to be highlighted that the participants did not require to do a specific pose for the tracking to start, like in the preliminary version of the tracking (Kinect for Windows SDK 1.0), but sometimes they had to move roughly to be detected as a human figure.

The optical tracking system was reported to be the most accurate solution by all the groups (*p* < 0.05), consistently with the results obtained in the study of the objective performance (in spite of the fact that the accuracy was analyzed in static condition) (Table 3. A3/B3 item). The same ranking order was found in the responses from healthy individuals and professionals. Individuals with stroke, however, reported the Kinect^TM^ to provide the lowest accuracy (*p* < 0.05). The presence of motor impairments could have led individuals with stroke to execute irregular movement patterns and postures while stepping that can affect the body parts recognition and skeleton fitting processes, thus affecting the scores of this tracking solution in the stroke group.

Similar conclusions can be inferred from the results of the robustness. In this case, all the groups defined the electromagnetic tracking solution as the most robust solution (*p* < 0.05), followed by the optical and the skeleton tracking system. Errors in the pose estimation could cause momentary maladjustments between the real and the virtual pose, which could be interpreted as a lack of robustness by all the participants, especially by individuals with stroke and professionals, who reported the lowest scores (3.4 ± 0.7 and 3.3 ± 0.8, respectively) (Table 3. A4/B4 item). The scores of the robustness were consistent with the jitter values derived from the study of the objective performance, indicating that this parameter could have been perceived as the most influential factor in the perception of robustness.

Healthy subjects and individuals with stroke evaluated the comfort of the three tracking systems (Figure 5). The skeleton tracking system was evaluated as the most comfortable solution by both groups (*p* < 0.001), followed by the optical and the electromagnetic solution (Table 3. A5 item). The absence of wearable devices in the skeleton tracking could have caused that this solution was perceived as the most comfortable configuration, while the use of sensors or markers could led to worse perceptions. Differences between the optical and the electromagnetic tracking systems were also reported by the healthy (*p* < 0.05) and stroke group (*p* < 0.001). While the optical solution only required participants to wear reflective markers attached to their ankles, the electromagnetic solution also required them to wear a hub held to the waist of their pants, which was connected through wires to the sensors. Even though the therapists placed the wires so that they did not interfere in the natural movement of the participants, both groups evaluated the electromagnetic tracking as the worst solution in terms of comfort.

Therapists were also asked about the ease of fixation of the markers/sensors, the insensibility of the performance to changes in the clinical setting, the ease of assistance, the maintenance of the tracking system, the working range of the tracking solution, the integration in the clinical setting, and the value for money of each tracking system (Figure 6). With regards to the ease of fixation, the professional group evaluated the skeleton tracking with the highest score, followed by the optical and the electromagnetic system (Table 3. B5 item). These results are consistent with the scores in the fixation speed. While the skeleton tracking did not use either markers or sensors, the other solutions required therapists to fix the markers in the ankle joint of the patients and the electromagnetic solution, in addition, required the fixation of the hub and the careful placement of the wires in order to avoid tangles. The time and ease of fixation are crucial factors that must be minimized in the clinic, where time is limited and should be dedicated to the physical therapy [29,30,31].

With regards to the sensibility to changes in the clinical setting, the therapists considered that the skeleton tracking system was the least susceptible system to changes of the environmental conditions in the physical therapy area (*p* < 0.001) (Table 3. B6 item), followed by the optical and the electromagnetic tracking systems. However, the overall scores were low in comparison with other items. The skeleton tracking system resulted the most robust tracking in presence of usual changes in the clinical environment. The optical solution, in contrast, was sometimes affected by the presence of reflections caused by chairs, room dividers, plinths, *etc.*, elements commonly present in the clinical setting, or even by the sunlight that went through the windows. Even though these issues can be avoided by removing these elements from the field of view of the cameras or by closing the blinds, physical therapy units are dynamic areas where the spatial distribution is constantly changing and the sunlight is appreciated. The electromagnetic tracking system proved to be the most susceptible solution to the environmental changes. As previously stated, ferromagnetic elements severely affected its performance. Even though some of them can be moved away, it was impossible to avoid the rebar of the floor.

However, the therapists reported that the electromagnetic tracking system was the solution that better allowed them to assist the patients, over the optical and skeleton tracking system. The physical principle of the G4^TM^ made the performance of the system possible even when the therapists were between the source and the sensors. It allowed them to freely assist the patients from any position, and even to manipulate their extremities if needed. The optical tracking, in contrast, required that the cameras had direct line-of-sight to the markers. This fact did not allowed the therapists to be in front of the patients, where they could occlude the markers. The assistance, although possible, had to be provided from behind. Similarly, the Kinect™ required direct line-of-sight with the participants. In this case, all their complete silhouettes had to be always present within the field of view of the camera. In addition, since the statistical method to detect the body segments was trained with isolated human poses, when therapists were close to the patients, manipulating or touching them, the system was not able to fit a skeleton in the resulting silhouette. Therapists had to hide from the field of view of the Kinect™ in order not to affect the tracking, which derived in significant lower scores (2.5 ± 0.9, *p* < 0.001) (Table 3. B7 item).

With regards to the maintenance, the therapists found that the need for recharging the hub of the electromagnetic tracking system after 7 to 8 hours of use was a limiting factor (*p* < 0.001) (Table 3. B8 item). The performance seemed to decrease as the battery got low. This was indicated by a flashing led on the hub. In order not to experience this effect during the therapy the clinicians recharged the hub by plugging it to the PC via USB connection or by using an AC adapter (fastest option). The other tracking solutions did not required special maintenance.

The professional group reported that the skeleton tracking system provided the largest working area, followed by the optical tracking system and the electromagnetic solution, which had significant lower scores (*p* < 0.05) (Table 3. B9 item). These scores were consistent with the experimental results. As previously stated, with our setting and independently of the accuracy, the Kinect^TM^ provided a working area of 3.1 m^2^, the optical solution provided an area of 2.6 m^2^, and the G4^TM^ provided an area of 2.2 m^2^ (Table 2).

Different factors that could affect the use of the tracking systems in physical therapy units were taken into account to evaluate the integration in the clinical setting. Therapists considered that the electromagnetic tracking system was the most likely to present problems in the clinical setting (*p* < 0.05). The high susceptibility of the G4^TM^ to ferromagnetic elements could have influenced its score (3.1 ± 0.6). The optical and skeleton tracking systems had higher but moderate scores (3.7 ± 0.5 and 4.2 ± 0.5, respectively) (Table 3. B10 item). The area needed by these solutions (3.75 × 1.5 m and 4 × 1.5 m) could have prevented higher scores, showing that this factor is critical to the integration in a physical therapy unit, where the space is often limited. Higher scores of the Kinect™ could be explained by a less sensitivity of this tracking system to environmental restrictions, such as reflections and sunlight, whose intensity and orientation vary all along the day.

The therapists were finally asked about the value for money of each system. The mass-produced Kinect, which had the lowest price, achieved the highest score (*p* < 0.001) (Table 3. B11 item). In addition, the Kinect^TM^ is an off-the-shelf device that is available worldwide. Scores to the other tracking solutions were also consistent with their price. However, though the price of the G4^TM^ was a few times higher than the price of the optical solution, the differences between these scores were not significant. This could be explained by a possible perception that both systems were less affordable to the same extent, besides their price.

Finally, all the groups were asked about their order of preference (Figure 7). The healthy group mostly preferred the Microsoft^®^ Kinect^TM^ (79.0%), over the other tracking systems, which is consistent with their scores to the comfort item (Table 3. A8/B12 item). These results can be explained by the fact that healthy individuals appreciated the comfort of this system over its worse accuracy or robustness. Remarkably, this group did not experience significant problems when interacting with the system and did not need assistance of the therapists, as the Kinect^TM^ is oriented towards healthy population. In contrast, the stroke group mostly preferred the optical tracking system (68.2%). The mentioned issues derived from a wrong skeleton fitting, more common in this group due to their motor restrictions, could have influenced their choice in the same way as it could have influenced their perceived success and agreement with the results. These facts should be specially taken into account when working with individuals with stroke, since they are likely to present behavioral problems [32], as irritability or depression, which can make this population particularly prone to frustration and reduce the benefits of rehabilitation [33]. In consequence, the use of the Kinect^TM^ as a tracking system for individuals with stroke could be restricted to subjects with specific motor conditions. The therapists mostly preferred the skeleton tracking (57.1%), slightly over the optical solution (35.7%). This result could be explained as a trade-off between both systems. In spite the ease and speed of the Kinect™ startup and its ease of maintenance, the aforementioned issues with the Kinect^TM^ can make the interaction of some patients difficult. The optical solution can overcome most of the interaction problems but it presents, however, some environmental restrictions (mainly light-related effects) that can affect their clinical use. It is important to highlight that, though the skeleton tracking system initially posed a challenge for the therapists, once they knew its functional limits, they were able to assist the patients. This fact, together with its affordable cost, could have led them to finally adopt the skeleton tracking solution over the other systems, and can be the reason why they are using it currently in their daily practice.

### 3.3. Limitations

The limitations of the study must be taken into account when interpreting its results. First, the characteristics of the stroke population are inherently linked to the specialized neurorehabilitation center where the study took place, which could restrict the generalizability of the results. Healthy subjects were chosen to match this population according to the inclusion/exclusion criteria. Second, the accuracy and jitter of the different tracking solutions were measured in static condition, which can noticeably change while moving. However, our results are also supported by previous studies that measure the accuracy in dynamic conditions [10]. Third, even though a cast was used to fix the foot, some instrumental error is expected to affect the measures. Finally, other interesting parameters are not measured in the study. For instance, early studies reported high latency in the Kinect^TM^ [12]. These values are even higher when using the Kinect^TM^ with custom-made applications on a PC, since the tracking processing has to be carried out by the CPU instead of by the GPU of the Microsoft^®^ Xbox 360^TM^ [7]. This could lead to noticeable delays between the real movement and their representation in the VE, thus affecting the feeling of presence [9] and the effect of the immediate feedback in the sensory integration [34].

## 4. Conclusions/Outlook

This paper studies and compares the performance of three different tracking solutions (optical, electromagnetic, and skeleton tracking) in a practical scenario and analyses the subjective perceptions of healthy and stroke individuals to the virtual experience when using the different technologies, and also their implications for therapists. Our results show that subjective perceptions and preferences are from being constant among different populations, thus suggesting that these considerations, together with the performance parameters, should be taken into account when designing a rehabilitation system.
